# Comparative study of acetylcholinesterase and glutathione S-transferase activities of closely related cave and surface *Asellus aquaticus* (Isopoda: Crustacea)

**DOI:** 10.1371/journal.pone.0176746

**Published:** 2017-05-09

**Authors:** Anita Jemec, David Škufca, Simona Prevorčnik, Žiga Fišer, Primož Zidar

**Affiliations:** University of Ljubljana, Biotechnical Faculty, Department of Biology, Ljubljana, Slovenia; Centre National de la Recherche Scientifique, FRANCE

## Abstract

The freshwater isopod crustacean *Asellus aquaticus* has recently been developed as an emerging invertebrate cave model for studying evolutionary and developmental biology. Mostly morphological and genetic differences between cave and surface *A*. *aquaticus* populations have been described up to now, while scarce data are available on other aspects, including physiology. The purpose of this study was to advance our understanding of the physiological differences between cave *A*. *aquaticus* and its surface-dwelling counterparts. We sampled two surface populations from the surface section of the sinking Pivka River (central Slovenia, Europe), i.e. locality Pivka Polje, and locality Planina Polje, and one cave population from the subterranean section of the sinking Pivka River, i.e. locality Planina Cave. Animals were sampled in spring, summer and autumn. We measured the activities of acetylcholinesterase (AChE) and glutathione S-transferase (GST) in individuals snap-frozen in the field immediately after collection. Acetylcholinesterase is likely related to animals’ locomotor activity, while GST activity is related to the metabolic activity of an organism. Our study shows significantly lower AChE and GST activities in the cave population in comparison to both surface *A*. *aquaticus* populations. This confirms the assumption that cave *A*. *aquaticus* have lower locomotor and metabolic activity than surface *A*. *aquaticus* in their respective natural environments. In surface *A*. *aquaticus* populations, seasonal fluctuations in GST activity were observed, while these were less pronounced in individuals from the more stable cave environment. On the other hand, AChE activity was generally season-independent in all populations. To our knowledge, this is the first study of its kind conducted in *A*. *aquaticus*. Our results show that among closely related cave and surface *A*. *aquaticus* populations also physiological differences are present besides the morphological and genetic. These findings contribute to a better understanding of the biology of *A*. *aquaticus* and cave crustaceans in general.

## Introduction

The freshwater isopod water louse, *Asellus aquaticus* (L.) (Isopoda: Crustacea), is a benthic crustacean with several important roles in freshwater ecosystems. It inhabits various freshwater habitats throughout most of Europe, including caves [1], [2]. The species exhibits strong genetic structuring in the southern and eastern part of its range [3], [4], [5]. Genetically distinct cave populations that resulted from polytopic and polychronous immigration to the cave environment still have their surface counterparts [4], [6], [7]. Pairs of surface and cave populations have gained increasing recognition as a model system to address questions of evolutionary parallelism and convergence [8], [9], [10].

The transition of organisms from surface to cave habitats presumably underlies adaptations at all levels of biological organization: morphology [9], [11], [12], [13], [14], physiology [15], [16], [17], [18], behaviour [16], [19], [20], and life histories [21], [22], [23]. Among these, mostly morphological [2], [24] and genetic differences [4], [6], [7], [8] between cave and surface *A*. *aquaticus* populations have been described, while scarce data are available on other aspects, including physiology. To our knowledge, only one physiological study comparing the cave and surface *A*. *aquaticus* has been carried out, and that was on respiration rate [25]. On the other hand, quite a few studies on presumable physiological adaptations of other cave crustaceans, such as different energy saving mechanisms, are available. These showed that many cave crustaceans have an amazing ability to endure starvation over prolonged periods of time due to their enhanced capacity for food storage [17], diminished locomotor activity [17], [18], and lower metabolic activity [16], [18], [26], [27], [28].

The purpose of this study was to advance our understanding of the physiological differences between the cave and surface *A*. *aquaticus*. While previous studies assessed the physiological state of some cave and surface crustaceans after a certain period of acclimation in the laboratory [18], [26], we measured the activities of two physiologically important enzymes in individuals snap-frozen immediately after collection. Acetylcholinesterase (AChE) plays a major role in cholinergic signal transmission in the sensory and neuromuscular systems and therefore appears to be likely related to animals’ locomotor activity [29], [30], [31]. Glutathione S-transferases (GSTs) are a family of multifunctional enzymes that play a central role in the detoxification of both endogenous and xenobiotic compounds and are also involved in intracellular transport, biosynthesis of hormones and protection against oxidative stress [32], [33], [34]. Some studies suggest that GST activity could be directly related to the metabolic activity of an organism. It is commonly assumed that higher metabolism leads to higher reactive oxygen species (ROS) production and results in higher GST activities [26], [35], [36], [37]. However, ROS levels and GST activities were also demonstrated to be increased under hypoxia (anoxia) and metabolic depression [38], [39], showing that the GST activity rate is not always representative of the basal metabolic rate.

The aim of this paper was to investigate whether AChE and GST activities differ between the cave *A*. *aquaticus* and its surface-dwelling counterparts, both living in well-oxygenated water. As cave animals are expected to have a reduced level of locomotor and metabolic activities, we hypothesized that generally there would be lower AChE and GST activities in cave compared to surface individuals. Due to more stable environmental conditions in caves, cave individuals were also expected to exhibit less season-dependent enzyme activities, in contrast to surface individuals, where considerable season-dependent enzyme activities were anticipated in accordance with evidence from studies on other crustaceans [40], [41].

## Materials and methods

### Chemicals

The following chemicals were purchased from Sigma (Germany): dibasic and monobasic potassium phosphate; 1-chloro-2,4-dinitrobenzene; L-glutathione (reduced form); 5,5-dithiobis(2-nitrobenzoic acid); sodium hydrogen carbonate; acetylthiocholine iodide. BCA Protein Assay Reagents A and B, cadmium chloride, and potassium dichromate were purchased from Pierce (U.S.A.). All chemicals were of the highest commercially available grade, typically 99% or higher.

### Study system and field work

**Fig 1:**
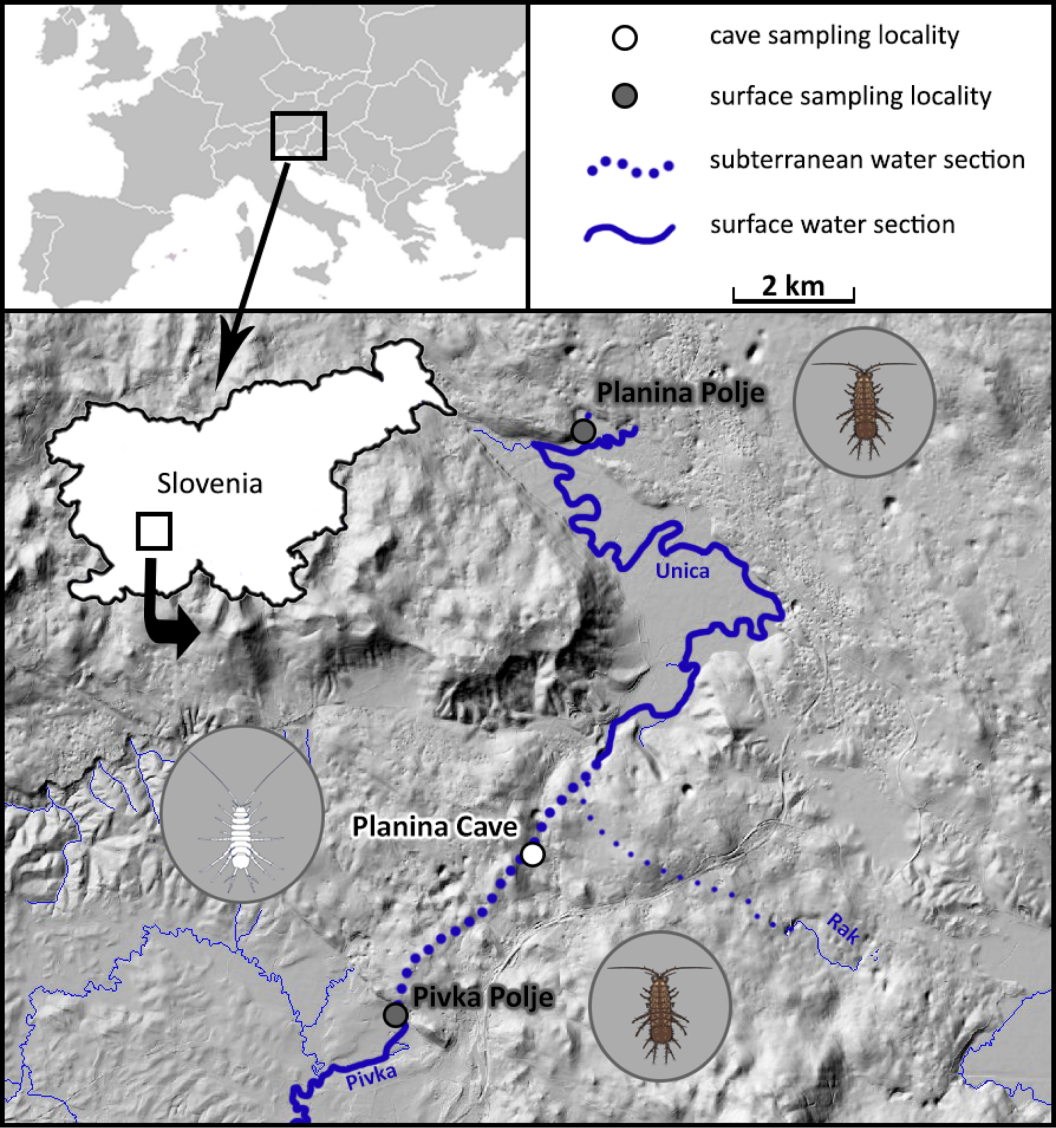
Sampling localities of *Asellus aquaticus*. (Digital elevation model is based on LIDAR (Light Detecting and Ranging) data freely available from ARSO—Slovenian Environment Agency at http://gis.arso.gov.si/evode/profile.aspx?id=atlas_voda_Lidar@Arso).

Closely related cave and surface populations of *A*. *aquaticus*, which inhabit subterranean and surface stretches of the sinking river Pivka (central Slovenia, Europe), provided an ideal ecological setup for the needs of our study. We sampled two surface populations from the river stretches flowing across two large enclosed karstic planes: (i) just prior to the river’s sink at Pivka Polje and (ii) after its resurgence on Planina Polje, and one cave population from (iii) the river’s subterranean section in the Planina Cave (Table 1, Fig 1). To account for seasonal differences in enzymatic activity, we conducted three samplings: spring, summer and autumn (Table 1). Each time 15 adult individuals regardless of sex, yet excluding ovigerous females, were collected from each population on the same day (hereafter referred to as a sample). Animals were snap-frozen on dry ice on site and immediately transferred to the laboratory where they were stored at -20°C until enzyme analysis. A reliable sex determination of *A*. *aquaticus* requires a careful examination of animal’s appendages (gonopods and peraeopod IV) under the stereo microscope. During this procedure we would risk that the specimens would warm up, which could endanger reliable enzyme measurements. We therefore did not determine the sex of each individual. During each sampling, we also measured physical and chemical properties of water at the sample site, i.e. temperature, dissolved oxygen concentration, conductivity, and pH, using a portable multimeter CyberScan 600 (Eutech Instruments).

**Table 1:** Information about sampling localities, taxa and dates of collection.

Locality	Slovene name	WGS84 coordinates	Taxon^1^	Ecomorph	Date of collection
Planina Cave^2^	Planinska jama	N 45.81990, E 14.24567^3^	*A*. *a*. *cavernicolus*	cave	spring: 18.3.2015
					summer: 17.7.2015
					autumn: 12.10.2015
Planina Polje	Planinsko polje	N 45.86872, E 14.24313	*A*. *a*. *carniolicus*	surface	spring: 18.3.2015
					summer: 17.7.2015
					autumn: 12.10.2015
Pivka Polje	Pivško polje	N 45.78137, E 14.20462	*A*. *a*. *aquaticus*	surface	spring: 18.3.2015
					summer: 17.7.2015
					autumn: 12.10.2015

^1^ Taxon names were assigned according to the currently valid taxonomy [1].

^2^ Two distinct cave populations of *A*. *aquaticus* inhabit the Planina Cave. In this study, the population from the Pivka River Channel, collected about 1 km from the cave entrance was used.

^3^ Coordinates for the cave locality were taken at the cave entrance.

### Enzyme analysis

Prior to enzyme analysis, the fresh mass of all individuals was measured (S1 Fig). Whole body homogenates were prepared in 650 μL of 50 mM phosphate buffer (pH 7.0) with 0.5% triton X100 using a T10 IKA Ultra-turrax homogenizer. The homogenate was centrifuged for 15 min at 16000 g and 4°C. Enzyme activities were measured on freshly prepared supernatants for each sample in triplicate.

AChE activity was determined according to the method of Ellman et al. [42] using microtiter plates as described by Jemec et al. [43]. Kinetic measurements were performed using acetylthiocholine iodide as a substrate (final concentration 1 mM). We mixed 1 M acetylthiocholine iodide and 2.3 mM 5,5-dithiobis(2-nitrobenzoic acid) in 1:500 (v/v) ratio. A total of 100 μL of this mixture was applied to the microtiter plate, where 50 μL of 50 mM potassium phosphate buffer (pH 7.0) and 50 μL of protein supernatant had already been added. The reaction was followed spectrophotometrically at 405 nm and 25°C for 5 min using a microplate reader (Anthos, UK).

GST activity was measured according to the method by Habig et al. [44] and optimized for microtiter plates [43] using 1,2-dichloro-4-nitrobenzene (CDNB) as a substrate. The final concentrations of substrates and reagents were: 4 mM of CDNB and 1 mM of reduced glutathione. CDNB was dissolved in ethanol and further diluted in 50 mM potassium phosphate buffer (pH 7.0) to final concentrations. The concentration of ethanol in the final reaction solution was less than 1% (v/v). We added 50 μl of the protein supernatant to start the reaction, which was followed spectrophotometrically at 340 nm and 25°C for 3 min using a microplate reader (Anthos, UK).

Protein concentration of the supernatants for enzyme analysis was measured using the BCA^™^ Protein Assay Kit, a modification of the bicinchoninic acid protein assay (Pierce, Rockford, IL, USA).

The AChE activity was calculated as nmoles of hydrolysed acetylthiocholine iodide/min/mg protein (extinction coefficient Ɛ_412_ = 13600 M^-1^cm^-1^) and GST activity as nmoles of hydrolysed CDNB/min/mg protein (extinction coefficient Ɛ_340_ = 9600 M^-1^cm^-1^). Hereafter, both of these are referred as an enzyme unit (EU). Protein concentration was calculated using bovine serum albumin as a reference.

### Data analysis

Data were statistically analysed in R 3.3.2 [45]. As variance was non-homogenous between groups, we employed a robust two-way ANOVA implemented in the R package WRS2 [46] with a pbad2way() function; a modified one-step M-estimator based on Huber’s Psi was used as a robust measure of central tendency (est parameter set to “mom”). Note that the M-estimators of the central tendency values were close to the mean values in all cases. A separate ANOVA model was run for each enzyme, i.e. AChE and GST. Enzyme activity served as a continuous response variable while population (Planina Cave, Planina Polje, Pivka Polje) and season (spring, summer, autumn) were included as categorical explanatory variables, whose interaction was tested as well. Biologically meaningful pairwise comparisons among groups were tested using the pb2gen() function and p-values were adjusted according to Benjamini & Hochberg [47]. Variability of samples was assessed using a robust measure of variability analogous to the standard deviation, i.e. median absolute deviation (MAD). Hereafter, we use the term significant difference to refer to statistically significant difference. All plots were drawn with OriginPro 8.0.

## Results

### AChE and GST activities

**Fig 2:**
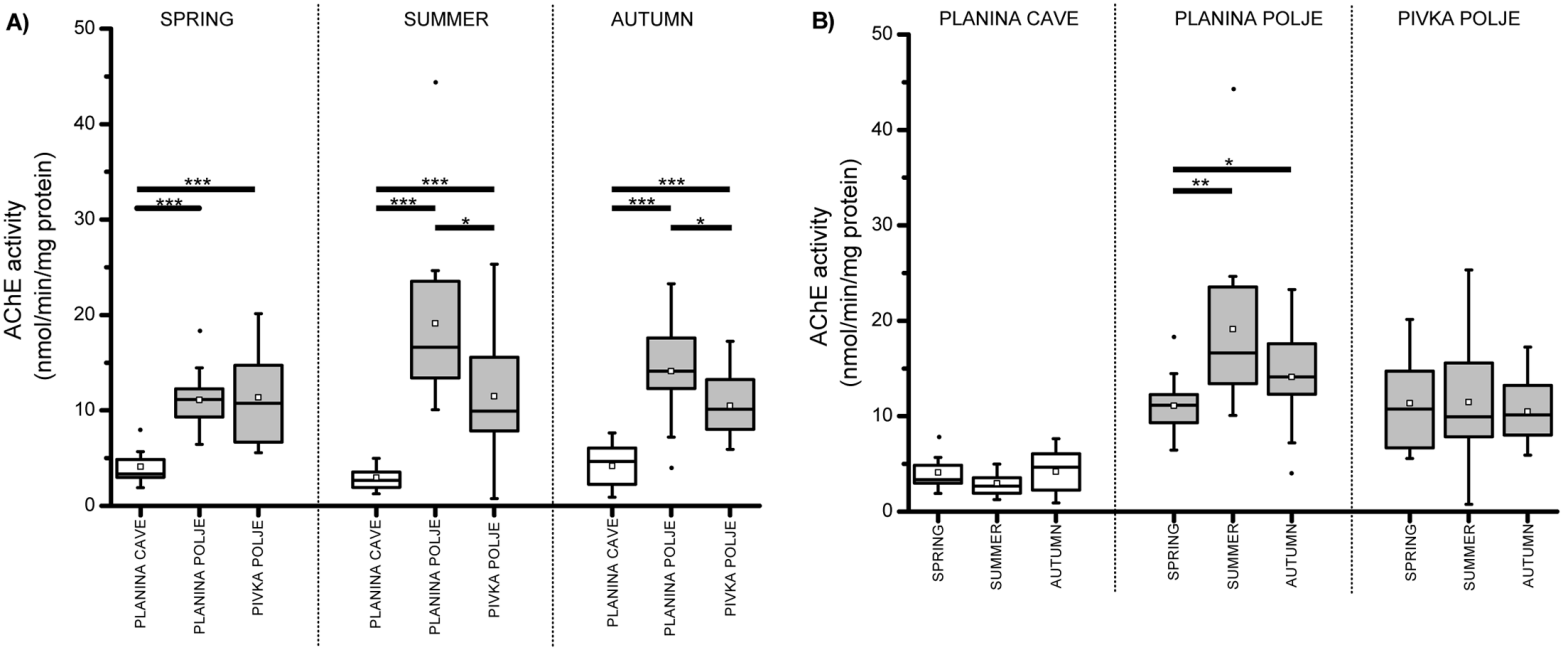
A) AChE activity in cave and surface *Asellus aquaticus* populations. B) Seasonal fluctuation in AChE activity in cave and surface *Asellus aquaticus* populations. Asterisks indicate statistically significant differences among samples (* 0.05 < p < 0.01, ** 0.01 < p < 0.001, *** p < 0.001). M-estimators of central tendency are shown as empty squares.

**Fig 3:**
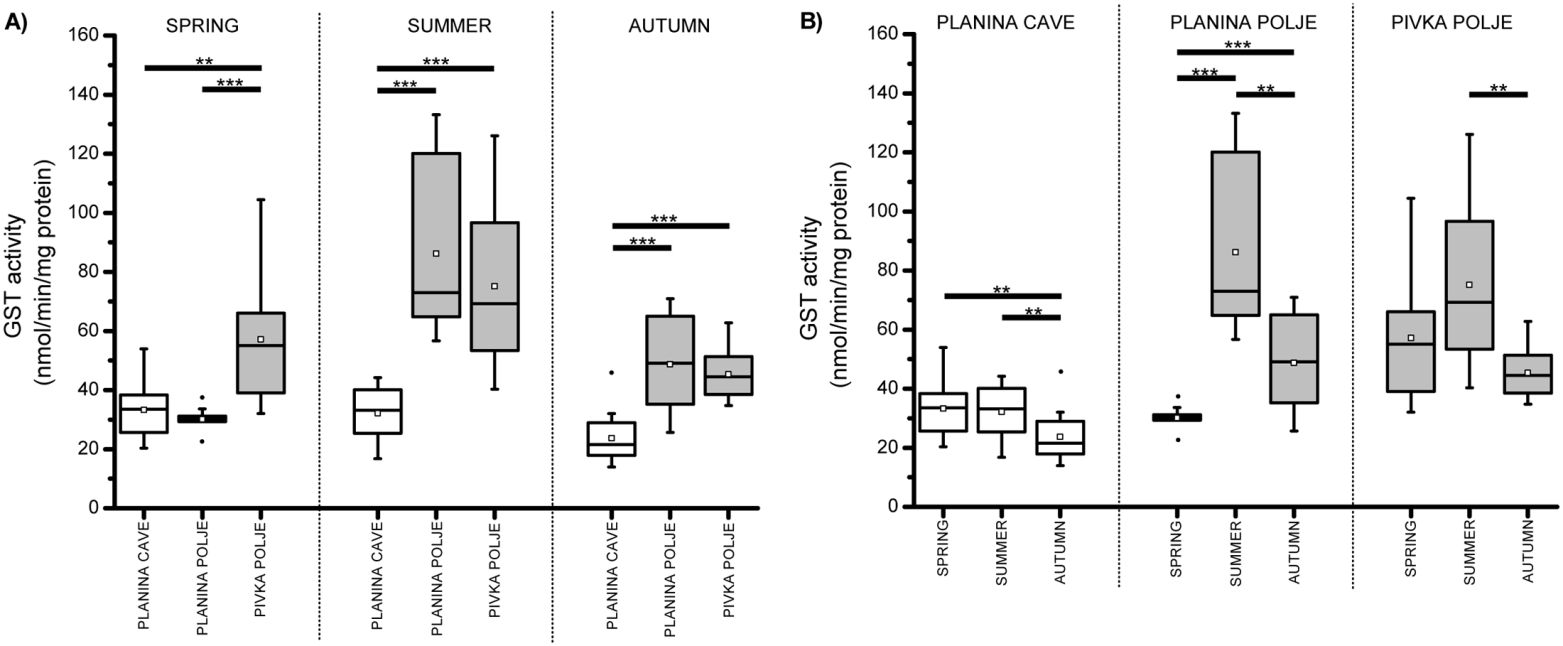
A) GST activity in cave and surface *Asellus aquaticus* populations. B) Seasonal fluctuation in GST activity in cave and surface *Asellus aquaticus* populations. Asterisks indicate statistically significant differences among samples (** 0.01 < p < 0.001, *** p < 0.001). M-estimators of central tendency are shown as empty squares.

Two-way robust ANOVA showed the same general pattern in AChE and GST: enzyme activities were noticeably lower in the cave population than in either surface population (Table 2, Figs 2A and 3A). Central values (see above) of AChE activity ranged between 3–4 EU in the cave population. In surface populations these values were 2.5 to 6-times higher, ranging between 10–11 EU and 11–18 EU in the Pivka Polje and Planina Polje populations, respectively. Similarly, central values of GST activity ranged between 23–33 EU in the cave population and were up to 2.5-times higher in both surface populations; between 45–75 EU and 30–80 EU in the Pivka Polje and Planina Polje populations, respectively (Table 2). Furthermore, between-individual variability in AChE and GST activity was generally lower in the cave population compared to either surface population during all seasons, with the exception of a slightlyhly variable GST activity in the spring sample from Planina Polje (S1 Table).

**Table 2:** Pairwise comparisons of AChE and GST activity among cave and surface *Asellus aquaticus* samples.

	AChE		GST	
**BETWEEN POPULATIONS/WITHIN SEASON**	**M-estimator**^1^ **[95% CI]**	**p-value**	**M-estimator**^1^ **[95% CI]**	**p-value**
Planina Cave (sp)–Planina Polje (sp)	-6.92 [-8.72, -5.24]	**< 0.001**	2.50 [-3.05, 8.07]	0.433
Planina Cave (sp)–Pivka Polje (sp)	-7.33 [-10.6, -3.78]	**< 0.001**	-22.99 [-36.08, -6.79]	**0.003**
Pivka Polje (sp)–Planina Polje (sp)	0.41 [-3.65, 3.62]	0.830	25.49 [9.87, 37.06]	**< 0.001**
Planina Cave (su)–Planina Polje (su)	-15.08 [-18.48, -11.24]	**< 0.001**	-47.07 [-71.76, -29.99]	**< 0.001**
Planina Cave (su)–Pivka Polje (su)	-8.35 [-11.89, -5.02]	**< 0.001**	-42.19 [-58.07, -19.19]	**< 0.001**
Pivka Polje (su)–Planina Polje (su)	-6.73 [-11.41, -1.85]	**0.021**	-4.89 [-41.87, 18.21]	0.777
Planina Cave (au)–Planina Polje (au)	-10.03 [-13.35, -7.23]	**< 0.001**	-26.09 [-38.91, -14.32]	**< 0.001**
Planina Cave (au)–Pivka Polje (au)	-6.14 [-9.1, -3.47]	**< 0.001**	-22.30 [-29.61, -14.21]	**< 0.001**
Pivka Polje (au)–Planina Polje (au)	-3.88 [-7.26, -0.77]	**0.033**	-3.79 [-16.49, 8.68]	0.518
**BETWEEN SEASONS/WITHIN POPULATION**	**M-estimator**^1^ **[95% CI]**	**p-value**	**M-estimator**^1^ **[95% CI]**	**p-value**
Planina Cave (sp)–Planina Cave (su)	1.13 [-2.37, 0.33]	0.231	0.38 [-7.69, 7.65]	0.935
Planina Cave (sp)–Planina Cave (au)	-0.23 [-2.3, 2.07]	0.834	10.14 [3.35, 17.91]	**0.012**
Planina Cave (su)–Planina Cave (au)	-1.36 [-3.24, 0.88]	0.457	9.75 [3.34, 17.77]	**0.009**
Planina Polje (sp)–Planina Polje (su)	-7.03 [-10.48, -3.07]	**0.001**	-49.20 [-73.13, -33.89]	**< 0.001**
Planina Polje (sp)–Planina Polje (au)	-3.33 [-6.19, -0.64]	**0.033**	-18.46 [-31.12, -6.23]	**0.002**
Planina Polje (su)–Planina Polje (au)	3.70 [-0.88, 7.52]	0.189	30.74 [11.94, 61.59]	**0.005**
Pivka Polje (sp)–Pivka Polje (su)	0.11 [-4.18, 4.87]	0.756	-18.82 [-40.41, 6.98]	0.262
Pivka Polje (sp)–Pivka Polje (au)	0.96 [-2.99, 4.54]	0.632	10.82 [-4.85, 24.72]	0.240
Pivka Polje (su)–Pivka Polje (au)	0.85 [-3.06, 4.84]	0.944	29.64 [7.08, 46.22]	**0.005**

^**1**^ Modified one-step M-estimator based on Huber’s Psi used as a robust measure of central tendency.

Bold and underlined text indicates statistically significant differences.

Season abbreviation: sp—spring; su—summer; au—autumn.

Pairwise comparisons (Table 2) confirmed that AChE and GST activities were significantly lower in the cave population compared to both surface populations in all seasons. Only the insignificantly different GST activities of the Planina Cave and Planina Polje populations collected in spring diverged from this pattern. The enzyme activities also differed between both surface populations, although not in all seasons and with a smaller effect size. AChE activity in the Planina Polje population was significantly higher in summer and autumn, while GST activity in the Pivka Polje population was higher in the spring.

According to the two-way robust ANOVA, seasonal fluctuation in enzyme activity in surface populations was observed, yet it did not follow any clear trend. The interaction between population and season was significant in both AChE (p = 0.012) and GST (p < 0.001), indicating that seasons had different effects on the enzyme activity of each population. For both enzymes this was mainly due to their more pronounced seasonal fluctuation in the Planina Polje population (see S2 Table for details). Pairwise comparisons revealed that seasonal fluctuation was more pronounced in the GST than in the AChE activity in all populations (Table 2, Figs 2B and 3B). The latter was almost constant in the Planina Cave and Pivka Polje samples, while it was significantly lower in spring in the Planina Polje population. GST activity in the Planina Cave population was significantly lower in autumn than in both other seasons while in the Pivka Polje population the low autumn value significantly differed only from the higher summer value. On the other hand, GST activity in the Planina Polje population was significantly lower in spring, as well as significantly higher in summer than in autumn.

### Physical and chemical parameters of water at sampling localities

**Fig 4:**
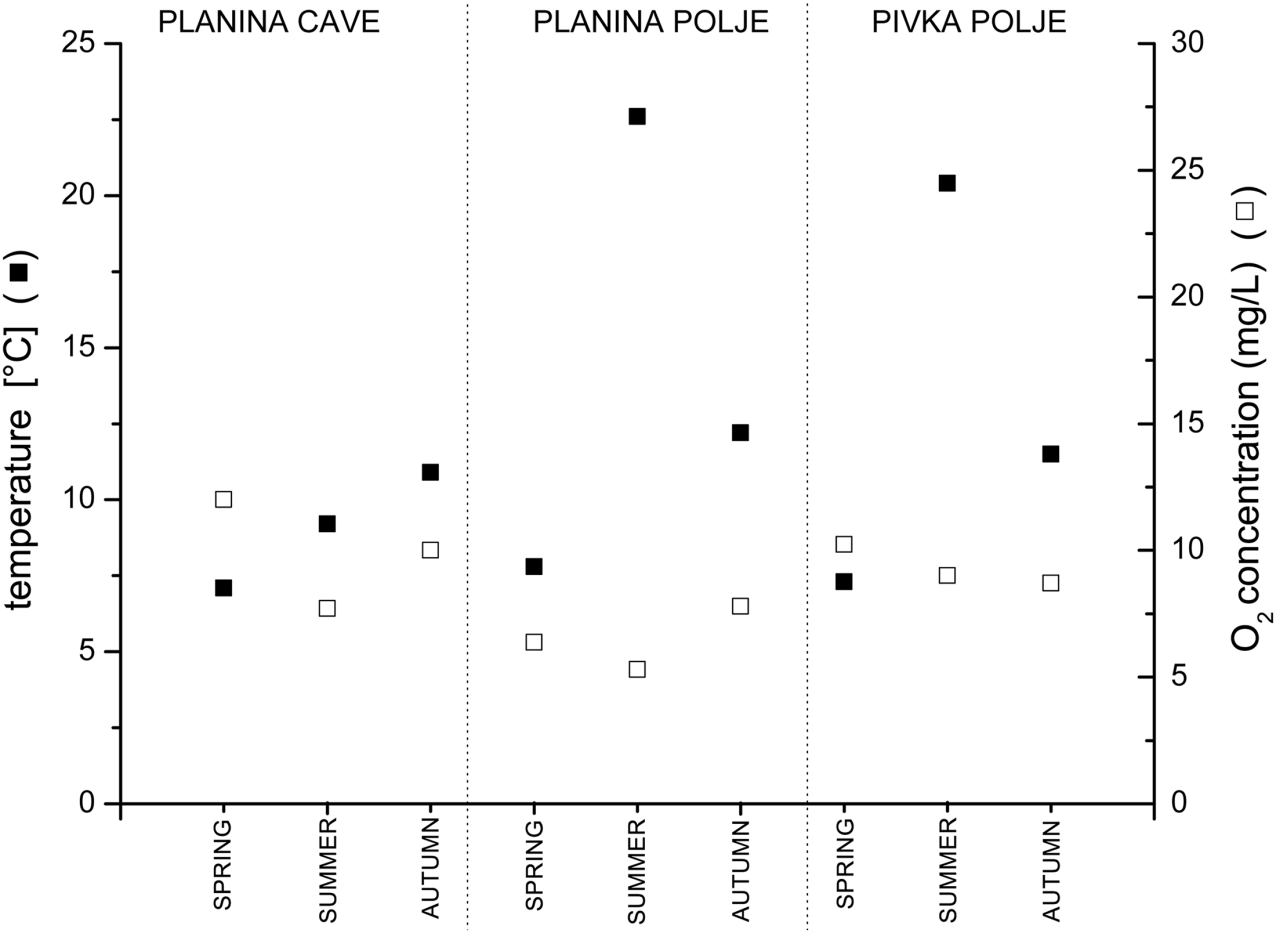
Water temperature and dissolved oxygen concentration at sampling localities.

Water temperature and dissolved oxygen concentration are shown in Fig 4, while all values of measured parameters are presented in S3 Table. As expected, variation in water temperature in the Planina Cave was negligible during the year. In spring and autumn, temperatures were similar at all three localities, while the summer temperatures at Pivka Polje and Planina Polje were considerably higher. Dissolved oxygen concentration was slightly lower in Planina Polje compared to Pivka Polje and Planina Cave, with mutually similar concentrations. Summer dissolved oxygen concentrations in Planina Cave and Planina Polje were lower than those measured in spring and autumn, while no seasonal fluctuation was observed in Pivka Polje. Water conductivity and pH were similar at all three localities throughout the year (see S3 Table).

## Discussion

Our study shows significantly lower AChE and GST activities in the cave population compared to both surface populations. These results support the suggested correlation between AChE activity and animals’ locomotor activity [29], [30] and the correlation between GST activity and animals' metabolic rate [36]. Namely, lower metabolic and locomotor activity of cave versus surface crustaceans has been reported in several comparative studies on locomotor [17], [18] and metabolic activities [16], [28], [48] of other cave and surface crustaceans. Decreased metabolic rates have also been shown in predatory stygobiotic amblyopsid fish compared to non-stygobiotic ones, presumably as a result of their adaptation to an energy-poor environment [49], [50].

Contrary, Mösslacher and Creuzé Des Châtelliers [25] observed higher locomotor activity and higher respiration rate in *A*. *aquaticus* from the chemoautotrophic Movile Cave (Romania) compared to surface individuals from Austria. However, the experimental design of their study was inappropriate to estimate animals’ locomotor activity since this behaviour was measured directly (i.e. by recording the time of movement), while animals simultaneously performed another type of behaviour (i.e. feeding on decaying leaves). Even the authors of the study have therefore acknowledged the high probability for biased results and speculative conclusions. Increased activity was also observed in characid *Astyanax* cavefish where it is a result of enhanced food searching behaviour [51], [52] and reduced sleep duration [53]. One has to consider though, that such behaviours likely provide an adaptive advantage to the predatory stygobiotic vertebrate, while it is harder to imagine their similar benefits for a grazing invertebrate such as *A*. *aquaticus*.

In surface *A*. *aquaticus* populations, evident seasonal fluctuations in GST activity were observed, while these were less pronounced in the cave population. There are a number of potential reasons for the observed results. Firstly, multiple environmental parameters, such as temperature [36], light intensity [27], [54], dissolved oxygen concentrations [38] and availability of food [17] are known to affect the GST activity of crustaceans. These environmental parameters are less variable in the stable cave environment compared to the surface [55], which may explain the lower GST activity fluctuation in cave individuals. Secondly, while mass of surface individuals differed significantly throughout the year, differences in mass were not as pronounced in the cave population (S1 Fig). In surface populations, we collected and compared three distinct generations over the three seasons [56], [57], [58], [59]. The overwintering generation included extremely large individuals collected in spring, the spring-brood generation included small individuals collected in summer, while a mixture of larger spring-brood and smaller summer-brood individuals was collected in autumn. Mentioned generations differ from one another according to reproductive stage, which is known to influence GST activity [60] via differences in metabolic activity. For example, it has been reported that the spring-brood generation in *A*. *aquaticus* matures within around 3 months, compared to the summer-brood generation that matures within about 6 months [58]. The described differences in life-histories are also the most plausible explanation for the single non-significant difference recorded between cave and surface populations used in our study, i.e. GST activity between the Planina Cave and Planina Polje spring sample. The overwintering individuals from Planina Polje were extremely large (S1 Fig) which may result in low GST activity as previously shown for other enzymes [41].

In contrast to the GST activity, the AChE activity at each locality did not vary throughout the seasons. There are mutually contradictory literature data for AChE dependence on environmental factors. While some authors report evident seasonal AChE fluctuation in crustaceans [40], [60], [61], [62], others state that some environmental parameters, such as temperature and salinity, do not influence AChE activity when studied in laboratory experiments [40], [63]. The only exception in our study was the surface population from Planina Polje, which had significantly lower AChE activity in the spring. The reason for this phenomenon remains unknown, but as already mentioned in the case of GST, we observed that the overwintering individuals at this locality were much larger and heavier than those from the other two seasons (S1 Fig). Similar observations were made by Xuereb et al. [41], where larger individuals of the freshwater amphipod *Gammarus fossarum* had a lower AChE activity than smaller ones.

Interestingly, larger variation in both enzyme activities was observed between surface than between cave individuals. Our study design does not allow the distinction between genetic and environmental causes for the observed differences. Nevertheless, the lower between-individual variability in the cave *A*. *aquaticus* may be due to strong directional and stabilizing natural selection for diminished locomotor and metabolic activity that supposedly increase fitness in an energy-poor environment. Just as likely, the higher variability between surface individuals could be due to the more variable environmental conditions at the surface. The same effect of the stable vs. changing environment on the variability in GST activities was observed in terrestrial isopods [43].

The employed enzymes are commonly used as biochemical biomarkers in routine environmental quality biomonitoring programmes [64], [65]. It has been already suggested that a number of environmental parameters and life-history traits of organisms could influence the enzyme activities [66]. The current study is an additional proof that the AChE and GST activities vary considerably with regard to locality and season, probably as a result of differences in environmental parameters and specific life-histories. Therefore, we suggest that potential future biomonitoring studies employing *A*. *aquaticus* should be designed closely in line with the results presented in this study.

To our knowledge, this is the first study of its kind conducted in *A*. *aquaticus*. In this model organism, AChE has so far been measured only as a biomarker of pollution in laboratory exposures [67], [68] and we found no records of GST measurements. Our future research will focus on verifying the actual correlation between both enzymes and their physiological roles. Confirmation of a direct link between *A*. *aquaticus* AChE and GST activity with its locomotor and metabolic activities would advance the use of biochemical approaches to studying both of these latter activities. The main advantage of such measurements is the assessment of the animals’ physiological condition *in-situ*, i.e. at the time and site of collection, as opposed to laboratory measurements that usually include an acclimation period. Namely, acclimation in the laboratory has been shown to alter the physiology of some crustaceans [43], [69].

**In conclusion**, our results show that considerable physiological differences exist between closely related cave and surface *A*. *aquaticus* populations. The lower AChE and GST activities of the cave population probably reflect physiological adaptations to the specific conditions of the cave environment. Seasonal fluctuation of GST enzyme activities was considerably more pronounced in surface populations and is likely a consequence of the joint effect of fluctuating environmental conditions and life histories. These findings contribute to a better understanding of both the biology of *A*. *aquaticus* and cave crustaceans in general.

## Supporting information

S1 Fig Seasonal fresh mass fluctuation in cave and surface *Asellus aquaticus* populations. (DOCX)

S1 Table Specific AChE and GST activities of cave and surface *Asellus aquaticus* samples. (DOCX)

S2 Table Specific AChE and GST activities. Robust two-way ANOVA post hoc tests comparisons of samples.
(DOCX)

S3 Table Physical and chemical parameters of water at sampling localities. (DOCX)
