# Supplementary material for: Comparative study of acetylcholinesterase and glutathione S-transferase activities of closely related cave and surface Asellus aquaticus (Isopoda: Crustacea)
Source: PLoS One. 2017 May 9;12(5):e0176746. doi: 10.1371/journal.pone.0176746 (PMC5423599; doi:10.1371/journal.pone.0176746)
Supplement: S1 Fig — (DOCX) [file pone.0176746.s001.docx]

**SUPPORTING INFORMATION**

COMPARATIVE STUDY OF ACETYLCHOLINESTERASE AND GLUTATHIONE S-TRANSFERASE ACTIVITIES OF CLOSELY RELATED CAVE AND SURFACE *ASELLUS AQUATICUS* (Isopoda: Crustacea)

Anita Jemec, David Škufca, Simona Prevorčnik, Žiga Fišer, Primož Zidar

University of Ljubljana, Biotechnical Faculty, Department of Biology, Jamnikarjeva 101, 1000 Ljubljana, Slovenia

**
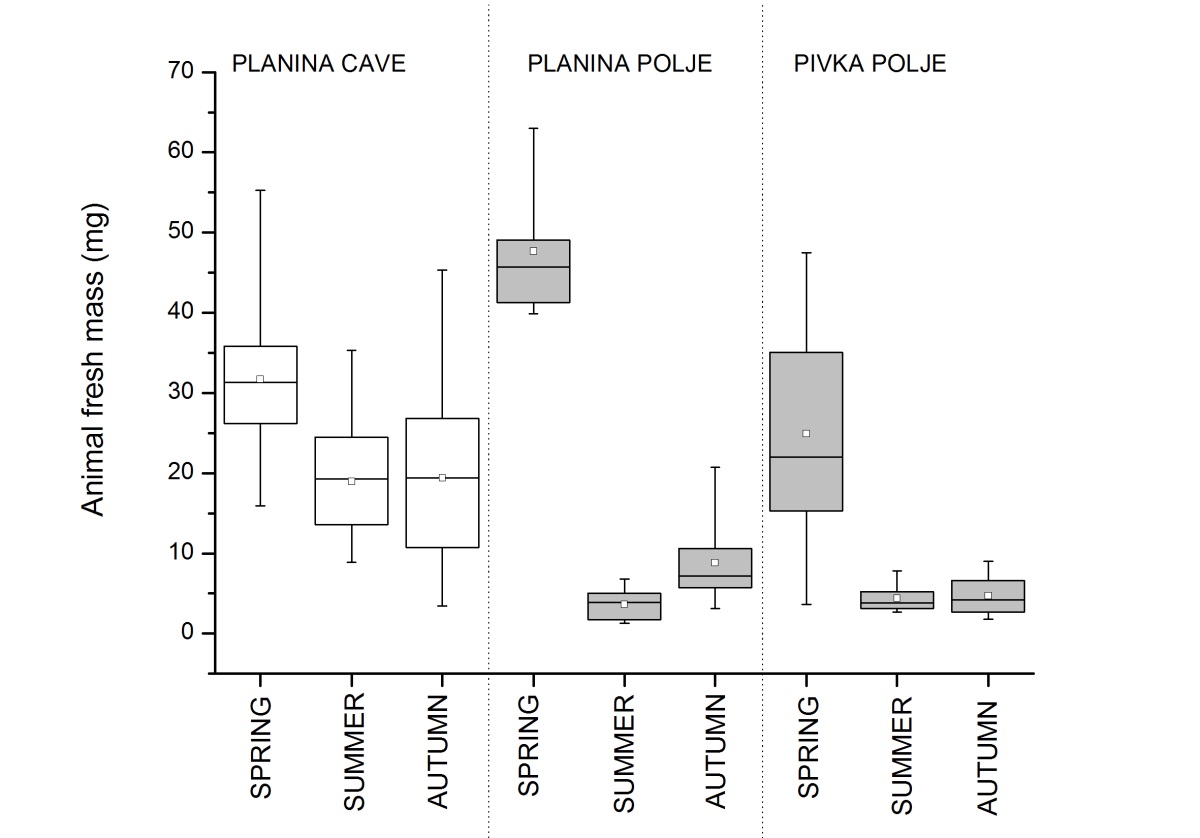
**

**Fig. S1.** Seasonal fresh mass fluctuation in cave and surface *Asellus aquaticus* populations. M-estimators of central tendency are shown as empty squares.
