# Supplementary material for: Comparative study of acetylcholinesterase and glutathione S-transferase activities of closely related cave and surface Asellus aquaticus (Isopoda: Crustacea)
Source: PLoS One. 2017 May 9;12(5):e0176746. doi: 10.1371/journal.pone.0176746 (PMC5423599; doi:10.1371/journal.pone.0176746)
Supplement: S1 Table — (DOCX) [file pone.0176746.s002.docx]

**SUPPORTING INFORMATION**

COMPARATIVE STUDY OF ACETYLCHOLINESTERASE AND GLUTATHIONE S-TRANSFERASE ACTIVITIES OF CLOSELY RELATED CAVE AND SURFACE *ASELLUS AQUATICUS* (Isopoda: Crustacea)

Anita Jemec, David Škufca, Simona Prevorčnik, Žiga Fišer, Primož Zidar

University of Ljubljana, Biotechnical Faculty, Department of Biology, Jamnikarjeva 101, 1000 Ljubljana, Slovenia

**S1 Table.** Specific AChE and GST activities of cave and surface *Asellus aquaticus* samples.

|  | **AChE** | | | **GST** | |
| --- | --- | --- | --- | --- | --- |
| **POPULATION (SEASON)** | **M-estimator^1^ [95% CI]** | **MAD/M^2^** | **M-estimator^1^ [95% CI]** | | **MAD/M^2^** |
| Planina Cave (spring) | 3.95 [3.13, 4.78] | 0.26 | 32.74 [27.49, 37.99] | | 0.16 |
| Planina Cave (summer) | 2.82 [2.13, 3.51] | 0.27 | 32.36 [27.33, 37.38] | | 0.21 |
| Planina Cave (autumn) | 4.18 [3.06, 5.31] | 0.42 | 22.60 [18.79, 26.42] | | 0.19 |
| Planina Polje (spring) | 10.87 [9.54, 12.21] | 0.11 | 30.24 [29.10, 31.37] | | 0.03 |
| Planina Polje (summer) | 17.90 [14.94, 20.87] | 0.25 | 79.43 [66.24, 92.63] | | 0.15 |
| Planina Polje (autumn) | 14.21 [11.85, 16.57] | 0.13 | 48.69 [40.65, 56.73] | | 0.28 |
| Pivka Polje (spring) | 11.29 [8.82, 13.75] | 0.37 | 55.73 [45.81, 65.64] | | 0.25 |
| Pivka Polje (summer) | 11.17 [8.32, 14.02] | 0.31 | 74.54 [59.46, 89.63] | | 0.31 |
| Pivka Polje (autumn) | 10.32 [8.67, 11.98] | 0.23 | 44.91 [40.21, 49.60] | | 0.13 |

**^1^** Modified one-step M-estimator used as a robust measure of central tendency and based on Huber’s Psi.

**^2^** Analog of the coefficient of variation used as a robust relative measure of variability, calculated as median absolute deviation (MAD) divided by median (M).
