# Supplementary material for: Comparative study of acetylcholinesterase and glutathione S-transferase activities of closely related cave and surface Asellus aquaticus (Isopoda: Crustacea)
Source: PLoS One. 2017 May 9;12(5):e0176746. doi: 10.1371/journal.pone.0176746 (PMC5423599; doi:10.1371/journal.pone.0176746)
Supplement: S2 Table — Robust two-way ANOVA post hoc tests comparisons of samples. (DOCX) [file pone.0176746.s003.docx]

**SUPPORTING INFORMATION**

COMPARATIVE STUDY OF ACETYLCHOLINESTERASE AND GLUTATHIONE S-TRANSFERASE ACTIVITIES OF CLOSELY RELATED CAVE AND SURFACE *ASELLUS AQUATICUS* (Isopoda: Crustacea)

Anita Jemec, David Škufca, Simona Prevorčnik, Žiga Fišer, Primož Zidar

University of Ljubljana, Biotechnical Faculty, Department of Biology, Jamnikarjeva 101, 1000 Ljubljana, Slovenia

**S2 Table.** Specific AChE and GST activities: Robust two-way ANOVA Post hoc tests comparisons of samples.

| **Comparison** | **AChE** | | **GST** | |
| --- | --- | --- | --- | --- |
|  | **M-estimator^1^ [95% CI]** | **p-value** | **M-estimator^1^ [95% CI]** | **p-value** |
| Planina Cave – Pivka Polje | -21.39 [-28.01, -15.59] | **< 0.001** | -88.25 [-110.26, -56.52] | **< 0.001** |
| Planina Cave – Planina Polje | -32.03 [-37.24, -26.58] | **< 0.001** | -64.47 [-109.67, -46.36] | **< 0.001** |
| Pivka Polje – Planina Polje | -10.64 [-17.79, -3.06] | **0.002** | 23.79 [-26.82, 48.74] | 0.214 |
| spring (sp) – summer (su) | -4.72 [-11.56, 1.05] | 0.066 | -63.03 [-105.51, -32.36] | **< 0.001** |
| spring (sp) – autumn (au) | -3.40 [-8.19, 2.91] | 0.159 | -0.36 [-16.61, 23.89] | 0.366 |
| Summer (su) – autumn (au) | 1.32 [-4.34, 8.61] | 0.235 | 62.67 [33.68, 109.44] | **< 0.001** |
| Planina Cave (sp – su) – Pivka Polje (sp – su) | -0.01 [-5.12, 7.40] | 0.476 | 20.86 [-13.53, 45.63] | 0.118 |
| Planina Cave (sp – au) – Pivka Polje (sp – au) | -1.27 [-6.39, 4.25] | 0.265 | 1.15 [-18.75, 21.35] | 0.487 |
| Planina Cave (su – au) – Pivka Polje (su – au) | -1.26 [-7.51, 3.60] | 0.266 | -19.71 [-41.05, 10.52] | 0.081 |
| Planina Cave (sp – su) – Planina Polje (sp – su) | 7.27 [2.40, 14.09] | **< 0.001** | 40.80 [27.74, 92.66] | **< 0.001** |
| Planina Cave (sp – au) – Planina Polje (sp – au) | 3.50 [-1.64, 7.29] | **0.038** | 28.08 [11.76, 47.52] | **< 0.001** |
| Planina Cave (su – au) – Planina Polje (su – au) | -3.76 [-11.10, 1.41] | **0.035** | -12.72 [-68.37, 6.78] | **0.034** |
| Pivka Polje (sp – su) – Planina Polje (sp – su) | 7.28 [-1.59, 13.99] | **0.013** | 19.94 [-7.14, 81.99] | **0.023** |
| Pivka Polje (sp – au) – Planina Polje (sp – au) | 4.77 [-1.79, 9.63] | **0.032** | 26.93 [6.45, 52.73] | **< 0.001** |
| Pivka Polje(su – au) – Planina Polje (su – au) | -2.50 [-11.02, 4.16] | 0.169 | 6.99 [-56.99, 32.32] | 0.487 |

**^1^** Modified one-step M-estimator used as a robust measure of central tendency and based on Huber’s Psi.

Bold and underlined text indicates statistically significant differences
