# Supplementary material for: Comparative study of acetylcholinesterase and glutathione S-transferase activities of closely related cave and surface Asellus aquaticus (Isopoda: Crustacea)
Source: PLoS One. 2017 May 9;12(5):e0176746. doi: 10.1371/journal.pone.0176746 (PMC5423599; doi:10.1371/journal.pone.0176746)
Supplement: S3 Table — (DOCX) [file pone.0176746.s004.docx]

**SUPPORTING INFORMATION**

COMPARATIVE STUDY OF ACETYLCHOLINESTERASE AND GLUTATHIONE S-TRANSFERASE ACTIVITIES OF CLOSELY RELATED CAVE AND SURFACE *ASELLUS AQUATICUS* (Isopoda: Crustacea)

Anita Jemec, David Škufca, Simona Prevorčnik, Žiga Fišer, Primož Zidar

University of Ljubljana, Biotechnical Faculty, Department of Biology, Jamnikarjeva 101, 1000 Ljubljana, Slovenia

**Table S3.** Physical and chemical parameters of water at sampling localities.

| **Locality and season** | **Temperature [°C]** | **O_2_ concentration [mg/L]** | **Conductivity [µS/cm^2^]** | **pH** |
| --- | --- | --- | --- | --- |
| Planina Cave |  |  |  |  |
| spring | 7.1 | 12.0 | 317.6 | 7.8 |
| summer | 9.2 | 7.7 | 250.7 | 8.0 |
| autumn | 10.9 | 10.0 | 251.7 | 7.7 |
| Pivka Polje |  |  |  |  |
| spring | 7.3 | 10.2 | 361.7 | 7.7 |
| summer | 28.0 | 10.5 | 286.4 | 8.5 |
| autumn | 11.5 | 8.7 | 233.5 | 7.7 |
| Planina Polje |  |  |  |  |
| spring | 7.8 | 6.4 | 230.2 | 7.3 |
| summer | 28.5 | 9.0 | 174.3 | 8.0 |
| autumn | 12.2 | 7.8 | 223.7 | 7.4 |
